# Supplementary figures and images for: Tulathromycin and Diclazuril Lack Efficacy against Theileria haneyi, but Tulathromycin Is Not Associated with Adverse Clinical Effects in Six Treated Adult Horses
Source: Pathogens. 2023 Mar 14;12(3):453. doi: 10.3390/pathogens12030453 (PMC10055745; doi:10.3390/pathogens12030453)

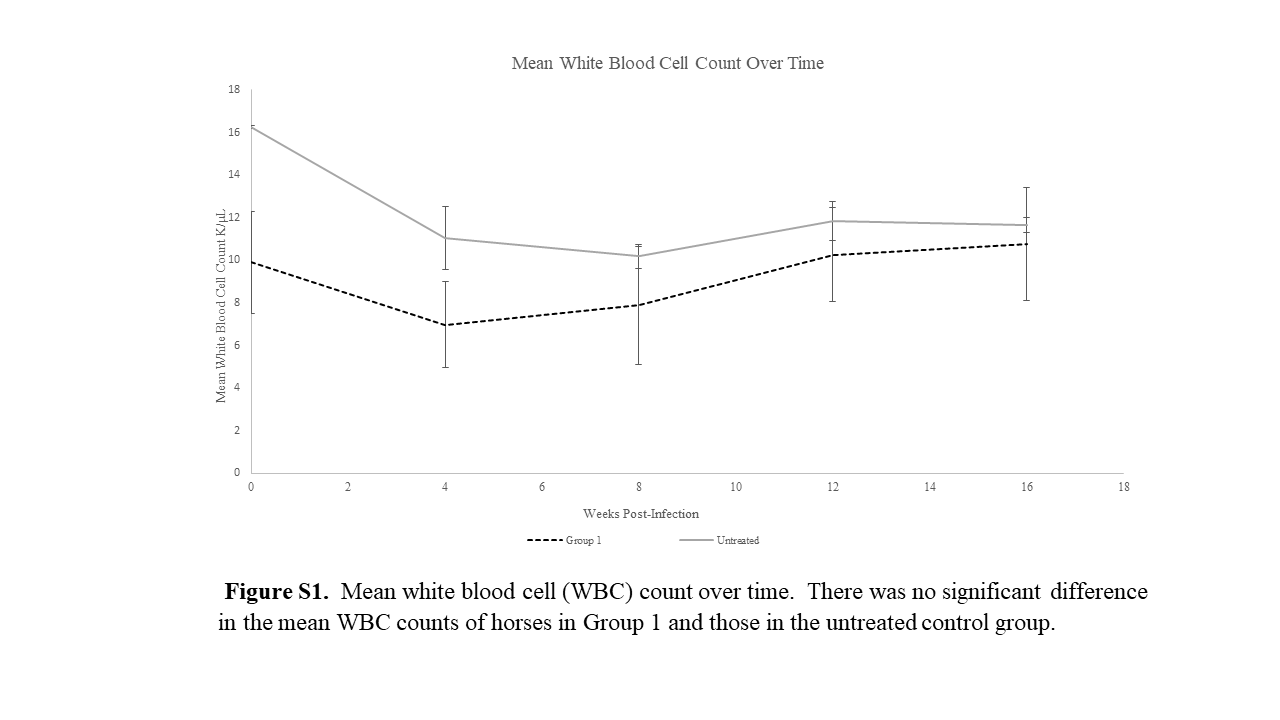

Supplement: Supplementary file 1 [file pathogens-12-00453-s001.zip › Figure S1.tif]

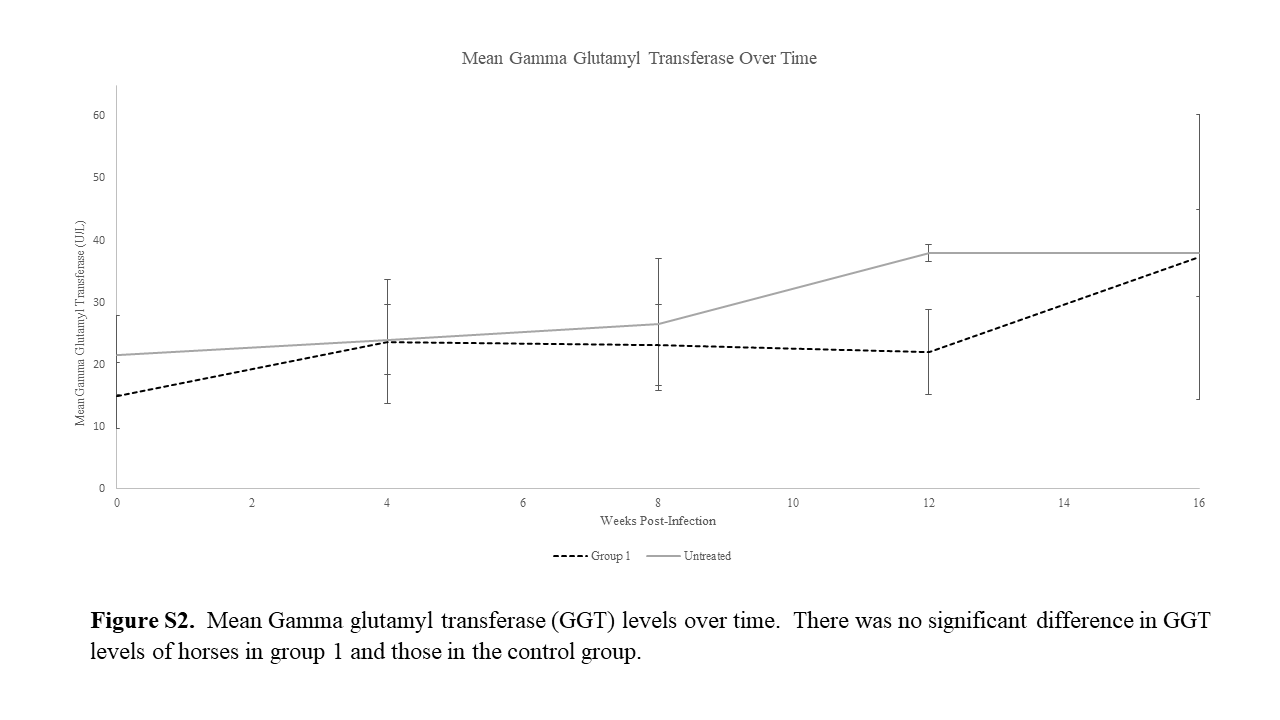

Supplement: Supplementary file 1 [file pathogens-12-00453-s001.zip › Figure S2.tif]

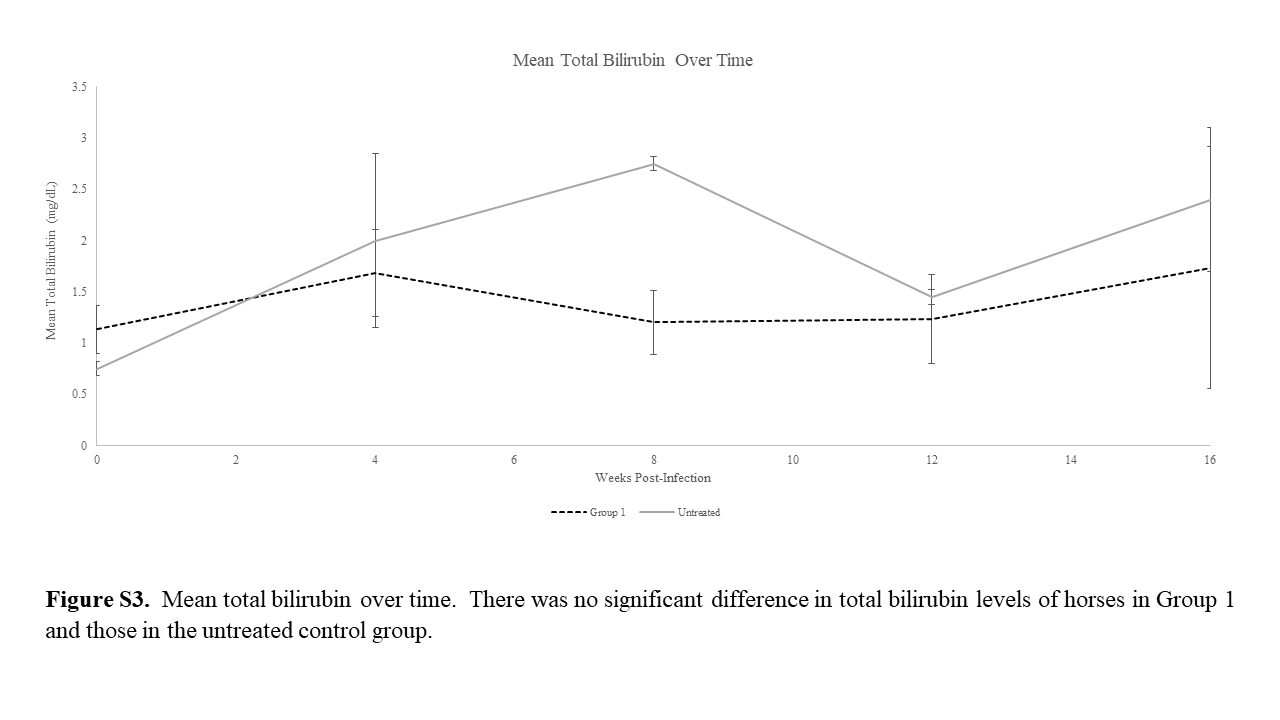

Supplement: Supplementary file 1 [file pathogens-12-00453-s001.zip › Figure S3.tif]

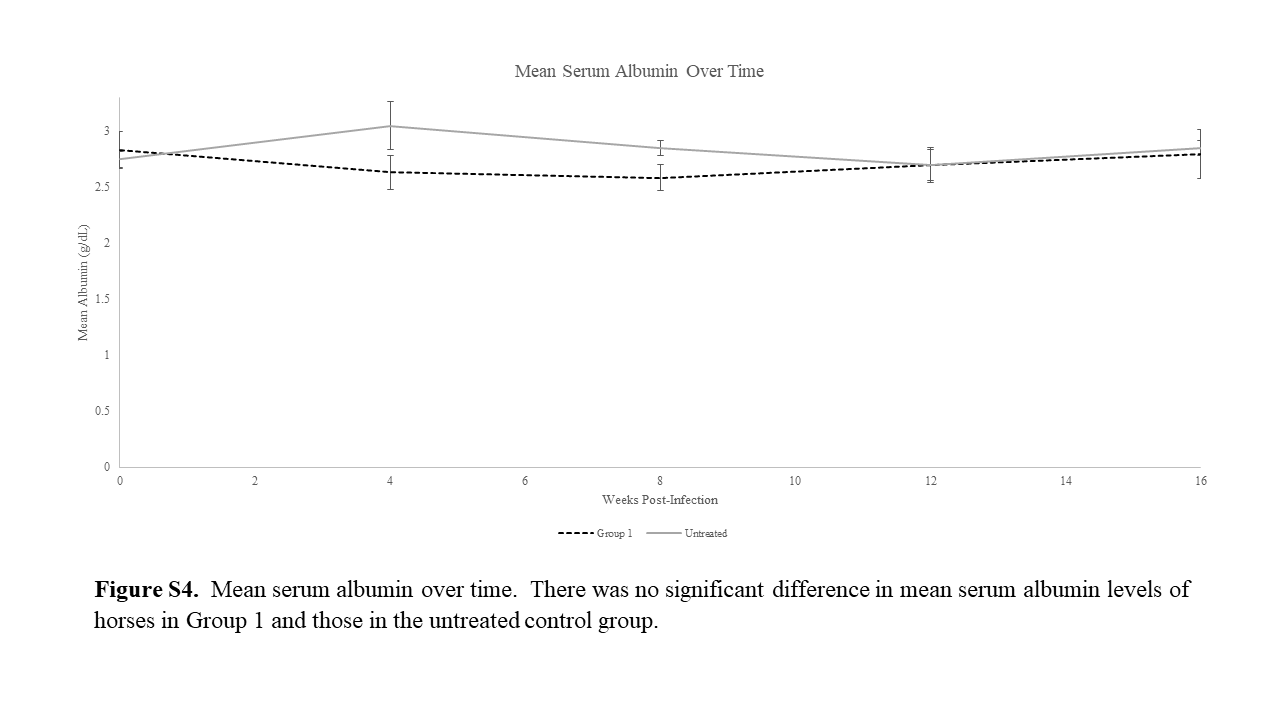

Supplement: Supplementary file 1 [file pathogens-12-00453-s001.zip › Figure S4.tif]

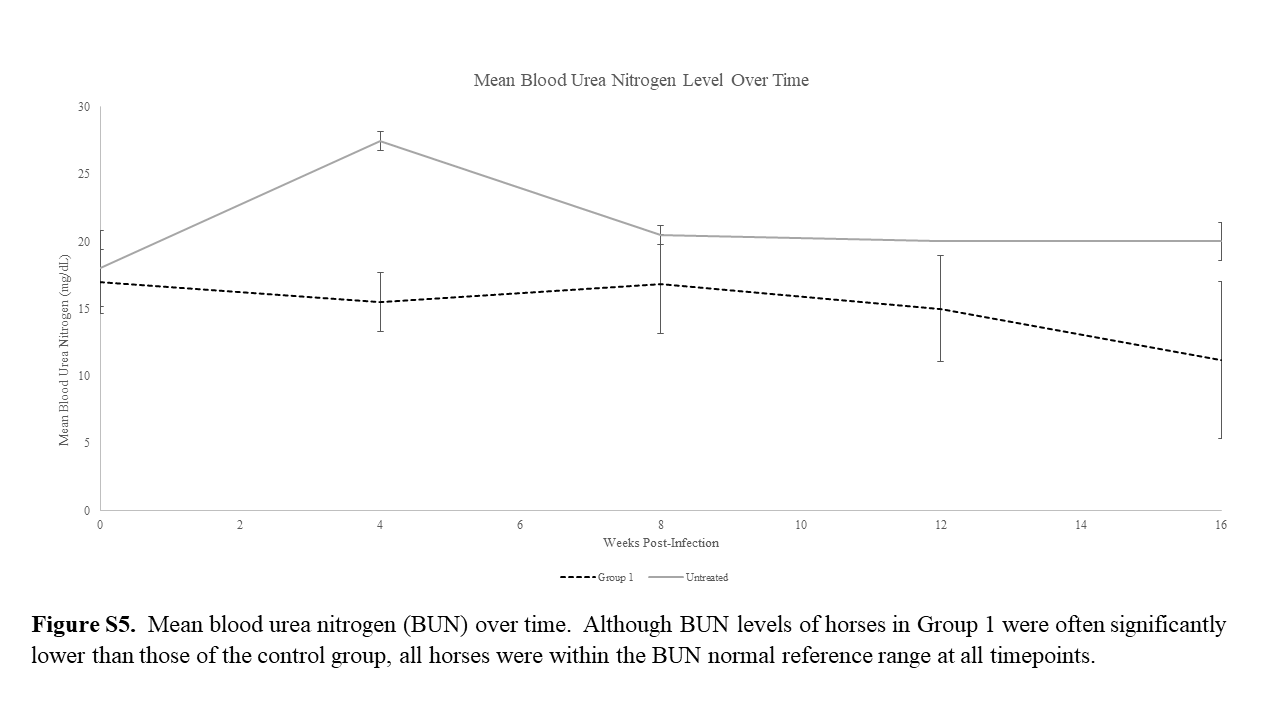

Supplement: Supplementary file 1 [file pathogens-12-00453-s001.zip › Figure S5.tif]

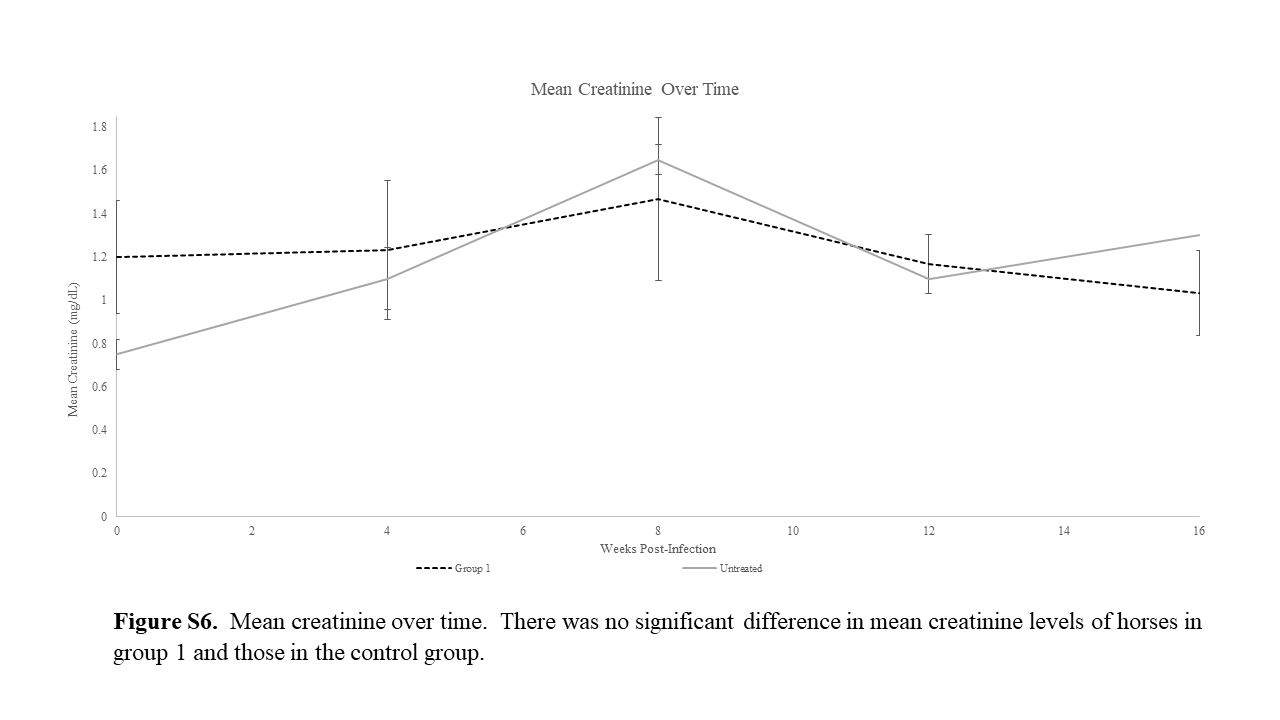

Supplement: Supplementary file 1 [file pathogens-12-00453-s001.zip › Figure S6.tif]

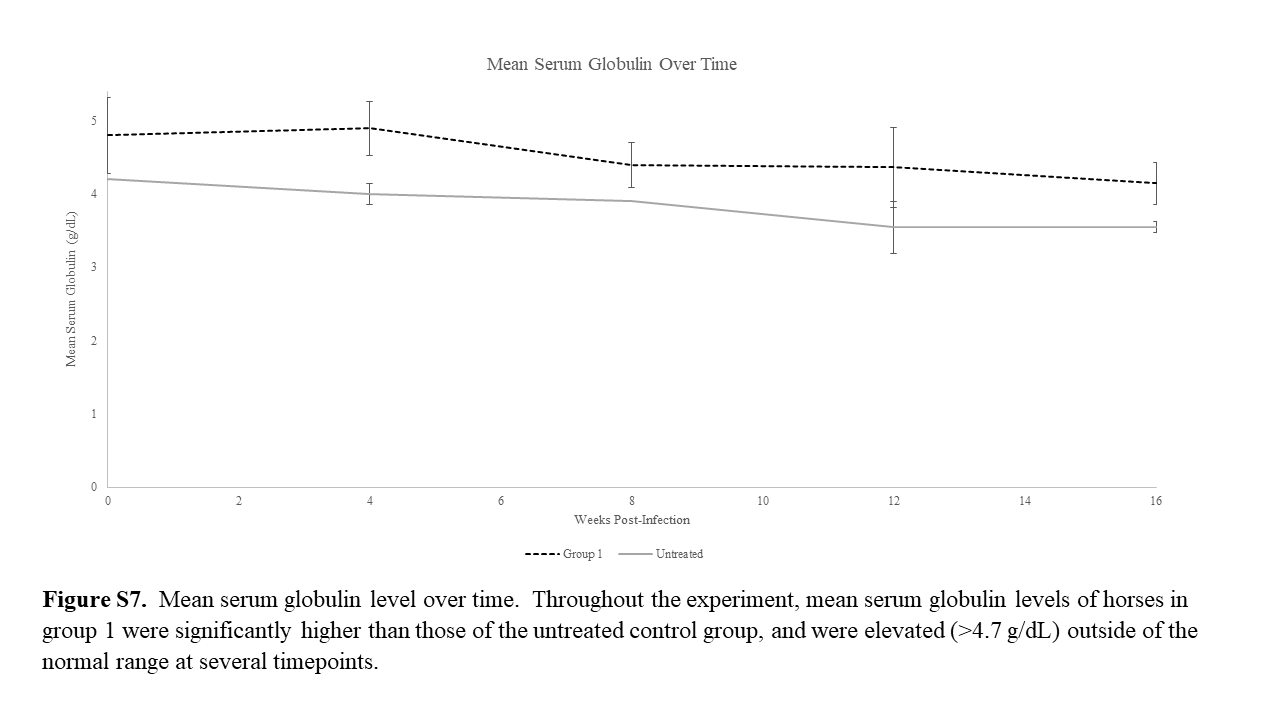

Supplement: Supplementary file 1 [file pathogens-12-00453-s001.zip › Figure S7.tif]

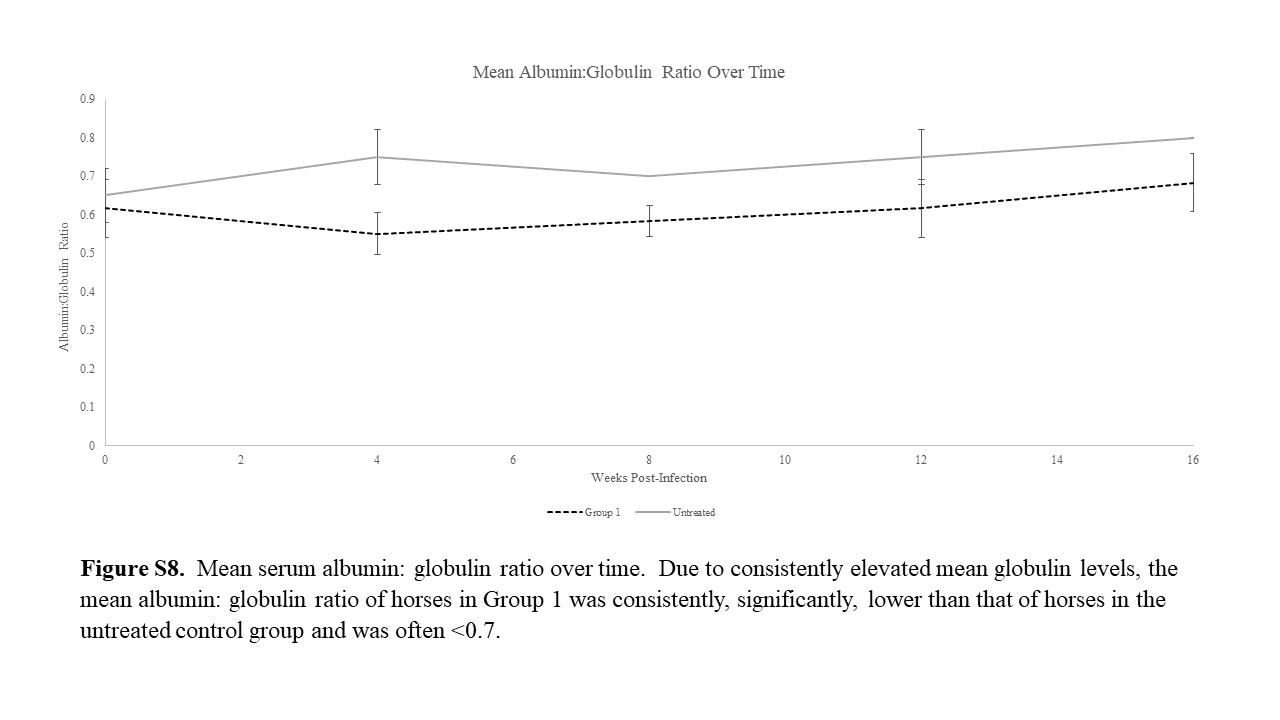

Supplement: Supplementary file 1 [file pathogens-12-00453-s001.zip › Figure S8.tif]
